# The emergence of pathogens on fish in an impacted estuary and the role of non‐native piranhas in a potential bacterial infectious outbreak

**DOI:** 10.1111/jfb.70377

**Published:** 2026-03-20

**Authors:** Ryan Andrades, Helder C. Guabiroba, Kathiani V. Bastos, Vítor L. A. Rodrigues, Marcelo R. D. Santos, Clarisse M. Arpini, Mayra Cunha Flecher, Helen A. Pichler, Ciro C. Vilar, Maurício Hostim‐Silva, Jean‐Christophe Joyeux

**Affiliations:** ^1^ Departamento de Oceanografia e Ecologia Universidade Federal do Espírito Santo Vitória Brazil; ^2^ Instituto Marcos Daniel Vitória Brazil; ^3^ Universidade Vila Velha Vila Velha Brazil; ^4^ Faculdade Brasileira Multivix Vila Velha Brazil; ^5^ Centro Universitário Norte do Espírito Santo Universidade Federal do Espírito Santo São Mateus Brazil

**Keywords:** fin rot, metal pollution, non‐native predators, opportunistic pathogens, Rio Doce, tropical ecology

## Abstract

As many tropical aquatic ecosystems worldwide, the Doce River estuary (DRE, southeastern Brazil) has increasingly faced multiple anthropogenic threats, including deforestation, mining, species introduction and water management. The 2015 collapse of the Fundão iron ore tailings dam severely changed water properties and increased heavy metal concentration in sediment. However, the potential synergistic effects of the observed threats on fish populations remain largely unknown. After detecting an elevated number of fin lesions in DRE fishes, we used the catfish *Genidens genidens* (Cuvier, 1829) to investigate whether such mutilations originated from fin‐nipping by non‐native piranha or emerged as a result of fin rot disease through bacterial infection. One third of the examined individuals had fin lesions in the DRE, whereas no conspecific exhibited such lesions in two control estuaries where piranhas are absent; lesions were restricted to the caudal fin, a region known to be targeted by fin‐nipping piranhas. Independently of the presence of fin lesion, culture of fins and livers indicated higher prevalence and variety of bacterial strains in DRE individuals than in a control area. Piranhas likely contribute to the fin lesions in DRE fishes. However, the higher prevalence and variety of bacterial pathogens as well as of liver and kidney disorders identified in fin‐healthy and mutilated fishes of the DRE suggest that the heavily metal‐polluted conditions in the DRE may be associated with the emergence of bacterial infections in fish.

## 1 INTRODUCTION

Tropical water bodies (e.g. rivers, lakes, estuaries and shallow reefs) hold most of the Earth's aquatic biodiversity, including 60%–80% of known freshwater and marine fish species (Barlow et al., 2018). However, these environments are under intense anthropogenic pressure due to habitat and species loss, climate change, resource overexploitation, species introduction and water pollution (Dudgeon et al., 2006; Halpern et al., 2008; Reid et al., 2019). In this context, biodiversity conservation in the tropics is especially complex due to the manifold environmental stressors, socio‐economic context and weak governance (Barlow et al., 2018; Connell & Hawker, 1992).

Inland and coastal South American aquatic habitats harbour almost 10,000 fish species, but well‐known stressors such as land use, dams, overfishing, mining and water pollution threaten this speciose fish fauna (Bezerra et al., 2019; Reis et al., 2016). Furthermore, the introduction of exotic species impacts the food‐web structure and induces a loss of native species, population declines through competition and predation and shifts in local fisheries (Alves et al., 2007; Fragoso‐Moura et al., 2016; Godinho et al., 1994; Latini et al., 2004; Pinto‐Coelho et al., 2008; Vieira, 2009). Invasive species are often tolerant to pollution (Karatayev et al., 2009), and the low‐health status of historically impacted environments, such as the Doce River basin (DRB, southeastern Brazil; see Espindola, 2015; Rodrigues et al., 2022), is expected to facilitate their success. For instance, carnivorous species of Serrasalmidae (known as piranhas) native from other drainages are massively abundant in the DRB, particularly in lakes adjacent to the river (Latini et al., 2004).

The DRB experienced one of the world's largest mining disasters in 2015: the Fundão dam collapse. The failure released ~43 million m^3^ of iron ore tailings into the DRB (Carmo et al., 2017). Seventeen days after the disaster, the contaminated mud reached the Doce River estuary (DRE) and the adjacent Atlantic coast. The mud consisted of silt, metals (Fe, Cd, Cr, Cu, Hg, Pb, Mn and Zn) and a metalloid (As) (Bianchini, 2016; Costa et al., 2022). A number of post‐disaster impacts on the estuarine biota have been detected (Andrades et al., 2020; Andrades et al., 2021; Gomes et al., 2017; Gabriel et al., 2020), and the persistence of contamination after years has resulted in chronic disturbances (Bernardino et al., 2019; Coppo et al., 2023).

Identifying the drivers of impacts on DRE fishes is a challenging task given that most drivers can act synergistically. For instance, disease emergence in aquatic habitats often is related to chemical pollution (Lindesjöö & Thulin, 1990; Vethaak et al., 1996; Vethaak & Rheinallt, 1992), and introduced species can trigger and, as optimal hosts, sustain diseases. In synergy, these impacts could jeopardize the populations of native and immuno‐depressed species (Poulin et al., 2011). In this context, the Guri sea catfish *Genidens genidens* (Cuvier, 1829) appears to be an appropriate native sentinel species for ecology and pollution investigations due to its high abundance and residency in the DRE and in other estuaries in the vicinity that can be used as control sites (Condini et al., 2021; Silva Junior et al., 2013; Vilar et al., 2022). In the DRE, *G*. *genidens* is often caught by hook‐and‐line and gillnets and represents an important fishery resource for subsistence and commercial purposes (Musiello‐Fernandes et al., 2024; Pinheiro & Joyeux, 2007).

**FIGURE 1:**
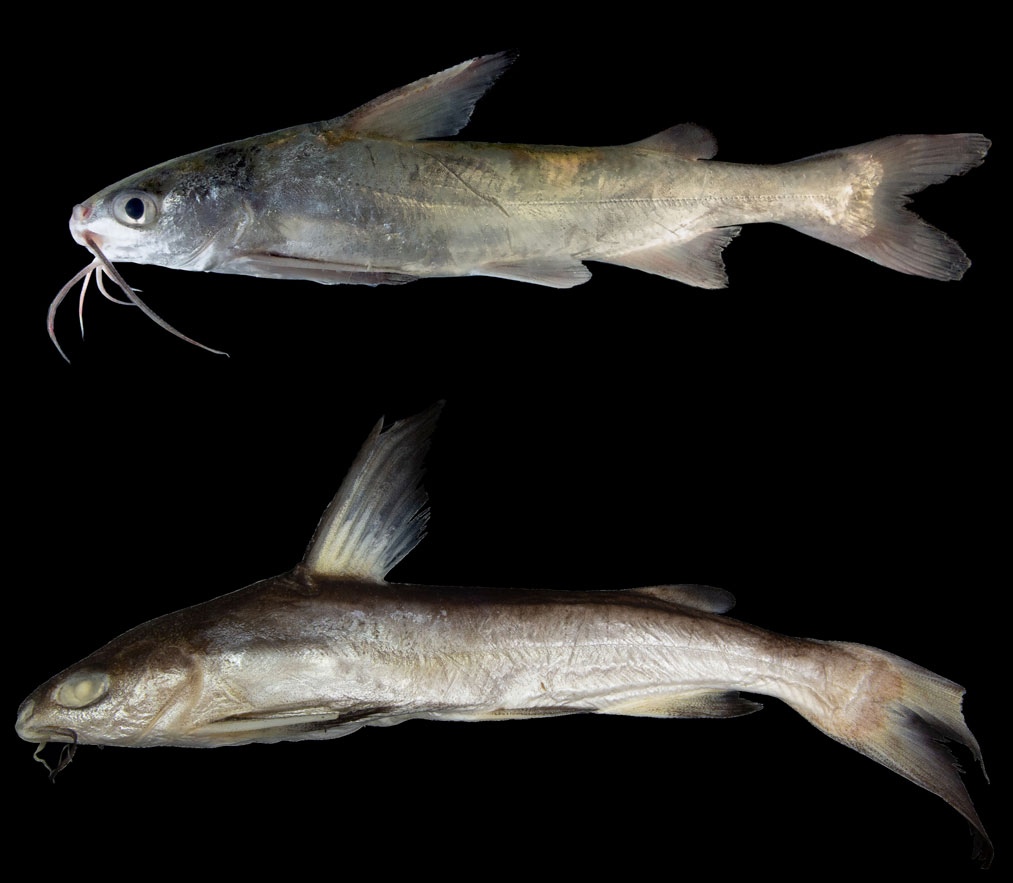
The catfish *Genidens genidens* collected in the Doce River estuary with no signs of fin lesions (above) and with caudal fin lesion (below). Photo: Flávio T. Szablak.

Recent fish sampling in the DRE for monitoring purposes has shown that fin lesions are common (Figure 1). These lesions could, hypothetically, be caused by piranha fin‐nipping (see Sazima & Machado, 1990; Silva et al., 2015), bacterial infection leading to fin rot disease or a combination of both. Fin rot is a bacterial disease characterized by a thickening of the fin epidermis, followed by necrosis of the soft tissue. Its aetiology is generally attributed to Gram‐negative bacteria (*Aeromonas*, *Pseudomonas* or *Vibrio*) (Austin & Austin, 2016; Bucke et al., 1996; Dar et al., 2022). Several studies have reported the occurrence of fin rot lesions in estuarine and coastal fishes in polluted environments (Dar et al., 2020; Mahoney et al., 1973; Mearns & Sherwood, 1974; Minchew & Yarbrough, 1977) and suggested the use of lesion appearance for aquatic pollution monitoring (Vethaak & Rheinallt, 1992). Here, we investigated two hypotheses (piranha attack and fin rot disease) on the origin of fin lesions using *G*. *genidens* as species model. Our aims are to investigate whether (1) piranha attacks are the primary source of fin injuries, which could subsequently become infected, or (2) pre‐existing bacterial infections (fin rot) are the main cause of fin tissue damage. As part of the investigation, we also assessed potential organ damage caused by bacterial infection in both lesioned and non‐lesioned fishes, as well as the prevalence of fin lesions in the DRE and two control estuaries.

## 2 MATERIALS AND METHODS

### 2.1 Study area and sampling design

The study was conducted in the DRE (19°39′ S, 39°49′ W) and in two control sites, all located in the state of the Espírito Santo, southeastern Brazil. The Doce River flows over 850 km from its source at 1200‐m elevation in the mountains of Minas Gerais state to the Atlantic Ocean. The climate near the mouth is tropical humid, and the rainy season extends from October to March (Oliveira & Quaresma, 2017).

We established as control sites the estuaries of the Itapemirim River (21°00′ S, 40°81′ W) and the São Mateus River (18°59′ S, 39°74′ W), where no piranha species has been recorded so far (Sarmento‐Soares & Martins‐Pinheiro, 2012, 2013) and whose estuarine fish communities have not been directly impacted by the tailings from the dam collapse. These control sites are located ~180 km to the south and 100 km to the north (in a straight line) of the DRE, respectively.

### 2.2 Specimen collection and processing

Specimens were obtained using a small‐sized otter trawl in January (Doce River), February (São Mateus) and May (Itapemirim) 2022. Water parameters (salinity, temperature and pH) were measured near the bottom using a Horiba U‐50 multiparameter at each site (see Condini et al., 2021). In the laboratory, *G*. *genidens* individuals were inspected for fin lesions independently by three researchers. We further selected subsamples to investigate whether the observed lesions were related to fin rot disease, that is a bacterial infection by *Aeromonas*, *Pseudomonas* or *Vibrio*. Thus, we built three groups of seven *G*. *genidens* each: group 1: DRE individuals with caudal fin lesioned; group 2: DRE individuals without caudal fin alteration; and group 3: control individuals sampled in the Itapemirim estuary.

Additional sampling was performed (June/July 2022) using the same gear to conduct a histopathological trial focused on kidney alterations caused by the systemic bacterial infections detected. We built three groups of 10 *G*. *genidens* each: group 1: DRE individuals caught in June 2022 with caudal fin lesioned; group 2: DRE individuals collected with group 1 but without caudal fin alteration; and group 3: control individuals sampled in the Itapemirim estuary in July 2022.

All *G*. *genidens* for bacterial and histopathological purposes were, immediately after collection, stored separately in plastic bags and transported to the laboratory in a cool reservoir to ensure thermonarcosis without freezing and then killed.

Additionally, we revisited voucher specimens at the Coleção Ictiológica da Universidade Federal do Espírito Santo (CIUFES) and other collected materials to determine the extent of fin lesions in the DRE fish community. Control sites are poorly represented at CIUFES and were not included in the search.

To investigate the potential relationship between fin lesion/mutilation and piranha attacks, we examined the stomach contents of individuals of two piranha species, *Serrasalmus* cf. *brandtii* Lütken, 1875, and *Pygocentrus nattereri* Kner, 1858, from the DRE. These individuals were collected by sieve and dip‐net samplings in 2019, 2021, 2022 and 2023. All piranha species individuals were stored separately in plastic bags after collection, frozen at −20°C and transported to the laboratory. In the laboratory, individuals had their stomachs removed for diet analysis under a stereomicroscope. Each prey item was identified, counted and weighed on a digital scale (Shimadzu AUW220D, 0.01 mg).

### 2.3 Bacterial analysis

In the laboratory, liver and caudal fin samples were extracted using sterilized apparatus. Liver samples were streaked on bacteriological media to ensure bacterial isolation. Culture on brain–heart infusion (BHI) agar for 24–36 h was prepared and posteriorly transferred to BHI agar plates with 5% sheep blood and MacConkey agar plates to promote the isolation of selective Gram‐negative bacteria. In addition, liver subsamples were ground, diluted in 0.85% saline solution and transferred to BHI agar plates with 5% sheep blood and MacConkey agar plates to ensure microorganism counting. Caudal fin samples were immersed in 70% alcohol solution for 1 min and washed with distilled water. Then, samples were cultured following the same protocol used for liver samples. All isolated samples were subjected to phenotypic characterizations (Table S1), and Gram‐negative bacteria were identified using Bactray Kit (Laborclin).

### 2.4 Histopathological analysis

Kidneys were extracted using sterilized apparatus. Kidney samples were fixed in 10% formalin solution and embedded in paraffin. Later, tissue fragments were processed using standard histological techniques, including dehydration in graded ethanol and clearing in xylene, followed by paraffin embedding; 4‐μm‐thick sections were obtained using a microtome and subsequently stained with haematoxylin and eosin for routine histopathological evaluation.

### 2.5 Statistical analysis

Analysis of similarities (ANOSIM) was applied to test whether bacterial strains differ among groups. Presence–absence matrices were constructed for fin and liver samples. For each matrix, the zero‐adjusted Bray–Curtis coefficient measure (in our case Sorensen distance) was calculated after the addition of a ‘dummy species’ to increase among‐sample data robustness (Clarke et al., 2006), because our dataset originally presented decreasingly sparse presence data. Differences in infectious load, expressed as the number of bacterial strains per individual, were tested among the three groups using a non‐parametric Kruskal–Wallis test (K–W). Post hoc pair‐wise Mann–Whitney (M–W) test was applied to detect the differences between pairs of groups. Both K–W and M–W tests results are based on 10,000 Monte Carlo resampling runs.

For kidney histopathological analysis, deviation from expected histopathological normality, that is, severity, was labelled as absent, mild, moderate or severe and correspondingly coded as 0, 1, 2 or 3. An overall score (histopathological concern) was prepared summing up all assessments for each individual. Note that the freshwater fish kidney may develop histological alterations in response to increasing osmotic demands (Hossain et al., 2022). However, the full spectrum of changes resulting from teleost estuarine residency is unclear (see Yancheva et al., 2016). We therefore follow Wolf et al. (2015) and refrain from interpreting kidney diagnosis in terms of organ damage or health status. Differences in infectious load in liver and in histopathological concern in kidney were tested among the three groups using non‐parametric K–W test. Post hoc pair‐wise M–W tests were applied to detect the differences between pairs of groups. Both K–W and M–W test results are based on 10,000 Monte Carlo resampling runs.

## 3 RESULTS

### 3.1 Fin lesions in DRE fishes: piranha attacks?

Stomach content analysis revealed that fins were the most frequent food item for *S*. cf. *brandtii* and that fish and insect provided the bulk of ingested biomass (Table 1). In contrast, *P*. *nattereri* stomachs mostly contained fish, either whole or as fish bones. Four individuals of this species had empty stomachs.

**TABLE 1:** Diet of two piranha species in the Doce River estuary.

Diet item	*Serrasalmus* cf. *brandtii* (*N* = 12)		*Pygocentrus nattereri* (*N* = 9)	
	W (g)	%FO	W (g)	%FO
Fish	0.04	8.3	8.67	55.5
Fish scales	<0.001	16.6	–	–
Fish fins	0.01	100	–	–
Insecta	0.03	8.3	0.03	11.1

Abbreviations: FO, occurrence frequency; W, wet weight of diet items.

### 3.2 Fin lesions in DRE fishes: fin rot disease?

**FIGURE 2:**
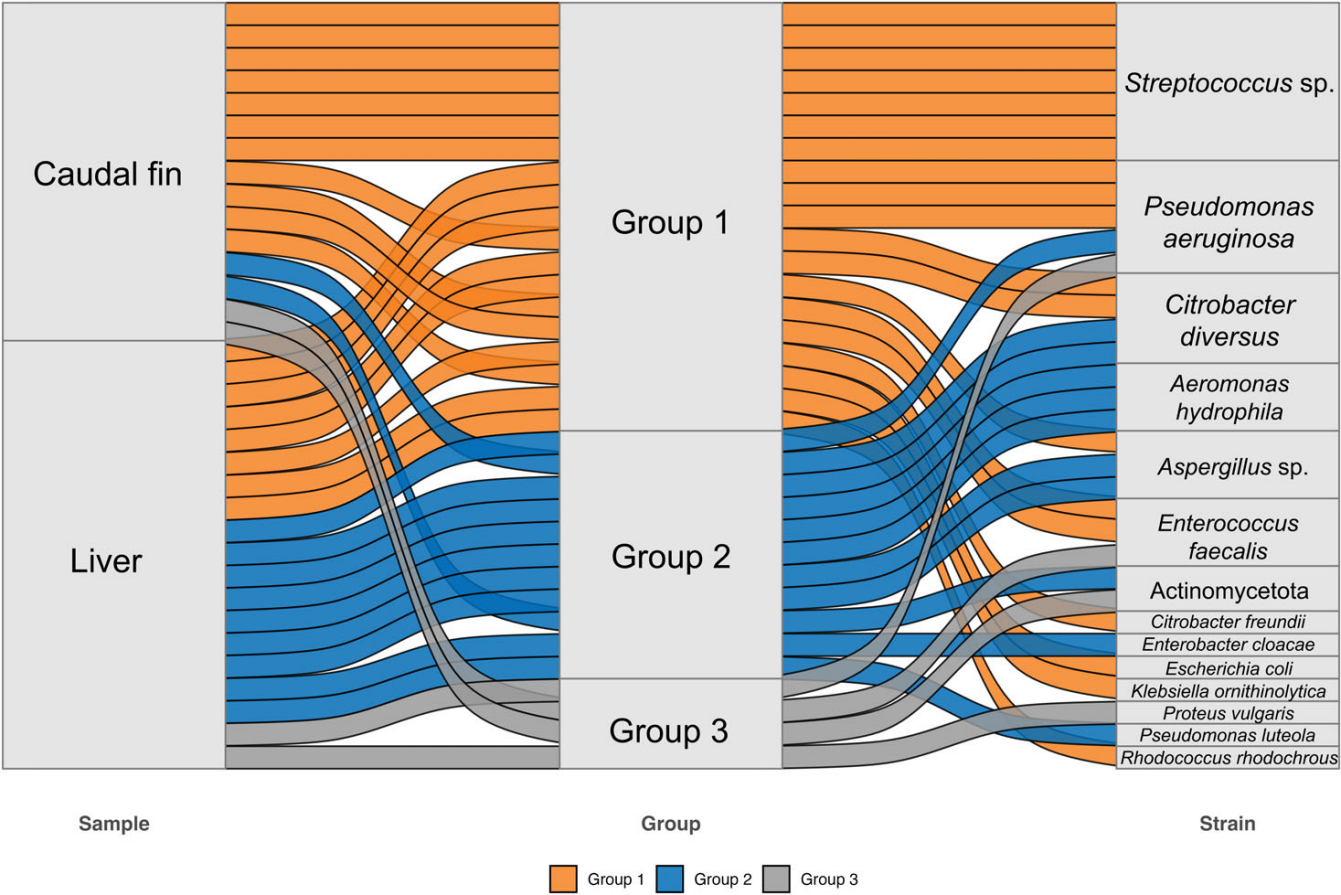
Bacterial (and fungal) strains isolated from caudal fin and liver samples of each group trial (*N* = 7 *Genidens genidens* individuals per group). Note that no bacterium or fungus was isolated from fin or liver of three individuals from group 3.

Phenotypic characterizations of isolated bacteria and one fungus (*Aspergillus* sp.) are presented in Table S1. Overall bacterial prevalence was higher in group 1 (DRE fish presenting with fin lesions) compared to groups 2 and 3 (left column in Figure 2). In group 1, isolated bacterial strains belonged to *Streptococcus* sp., *Citrobacter diversus*, *Escherichia coli* and *Enterococcus faecalis*. Prevalence was 100%; that is, all caudal fins were contaminated. In group 2, *C. diversus* and a non‐identified Gram‐positive bacterium (Actinomycetota) were isolated, whereas in group 3, *E. faecalis* and Actinomycetota were isolated (Figure 2). Prevalence in groups 2 and 3 was 29% (two of seven specimens). ANOSIM showed that different sets of bacteria infected the three groups (*p* < 0.01, *R* ^2^ = 0.5), with a greater strain variety in group 1.

In liver, bacterial and fungal strains isolated from group 1 were *Pseudomonas aeruginosa*, *Klebsiella ornithinolytica*, *Aspergillus* sp., *Rhodococcus rhodochrous*, *C. diversus* and *Citrobacter freundii*. Prevalence was 71.4% (five out of seven individuals). In group 2, *Aeromonas hydrophila*, *Aspergillus* sp., *C. diversus*, *Enterobacter cloacae*, *Pseudomonas luteola* and *P. aeruginosa* were isolated with a prevalence of 86% (six out of seven individuals). In group 3, overall prevalence of *P. aeruginosa* and *Proteus vulgaris* was 29% (two out of seven individuals; Figure 2). ANOSIM indicated that bacterial strains isolated from the liver of groups 1 and 2 were marginally dissimilar (*p* = 0.07, *R* ^2^ = 0.1) to those in group 3.

Infection or coinfection varied widely among groups; the number of bacterial strains per individual fish (independent of organ/structure) was higher in group 1 [mean ± standard deviation (SD): 2.71 ± 1.11, range = 2–5], intermediate in group 2 (mean ± SD: 1.57 ± 0.79, range = 1–3) and lowest in group 3 (mean ± SD: 0.57 ± 0.53, range = 0–1). Thus, the number of strains varied among groups (K–W: *χ* ^2^ = 13.273, *p* < 0.001). Post hoc M–W tests evidenced that all groups differed from each other (groups 1 and 2: *Z* = −2.097, *p* = 0.038; groups 1 and 3: *Z* = −3.220, *p* = 0.001; groups 2 and 3: *Z* = −2.351, *p* = 0.043).

**FIGURE 3:**
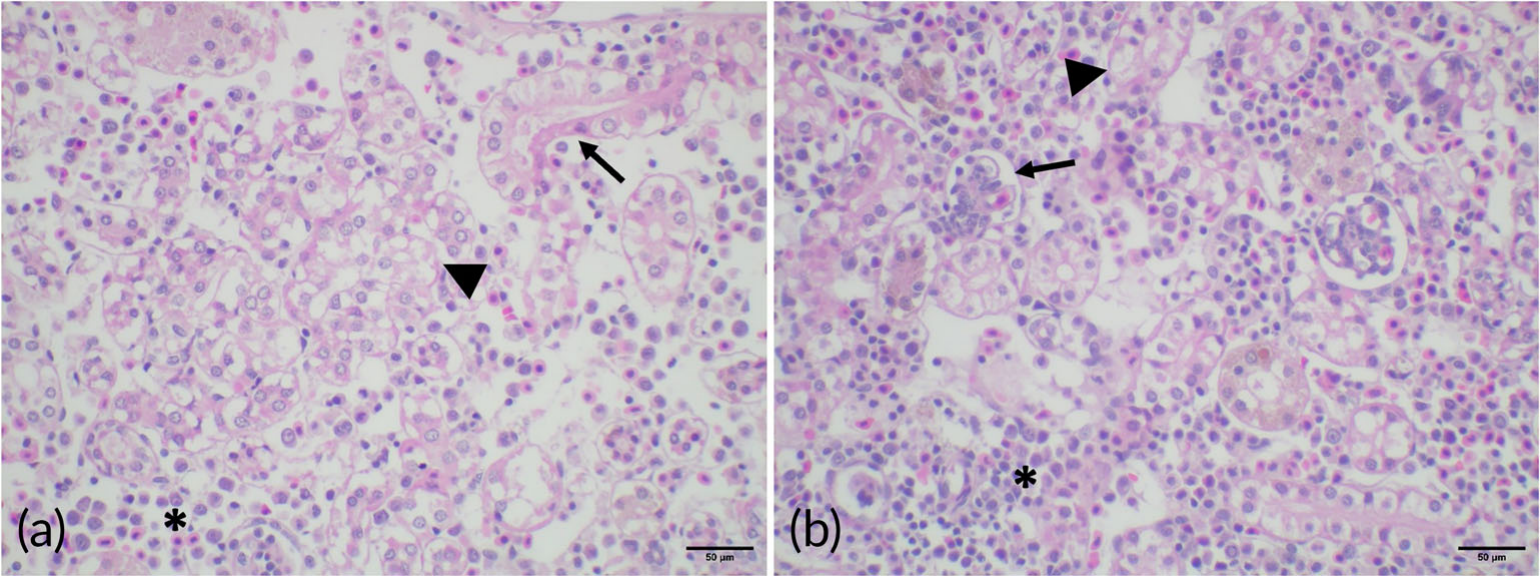
Fish kidney microscopic image. (a) Renal tubule cells showing pronounced vacuolization of renal tubular epithelium (arrowhead), multifocal necrosis (arrow) and moderate interstitial nephritis (asterisk). H&E (haematoxylin and eosin), 40×, scale bar = 50 μm. (b) Renal tubule cells showing mild vacuolization of renal tubular epithelium (arrowhead), glomerular atrophy (arrow) and pronounced interstitial nephritis (asterisk). H&E, 40×, scale bar = 50 μm.

### 3.3 Fin lesions in DRE fishes: internal infection and histopathological concern

Liver infection, as detected by culture growth, was widespread (90% of individuals exhibited bacterial growth) and strong in group 1 (mean ± SD: 4.90 ± 3.96 μfc/g, range = 0–5.33) but sporadic (40% and 60%) and weak in groups 2 and 3, respectively (0.63 ± 1.07 μfc/g, range = 0–3.33; 1.73 ± 2.24 μfc/g, range = 0–5.66, respectively). Bacterial counts differed among the three groups (K–W: *χ* ^2^ = 9.173, *p* = 0.007), and post hoc M–W tests evidenced that lesioned DRE fishes of group 1 exhibited stronger hepatic infections than non‐lesioned specimens of DRE and control specimens of Itapemirim (groups 1 and 2: *Z* = −2.861, *p* = 0.004; groups 1 and 3: *Z* = −2.020, *p* = 0.043). Groups 2 and 3 exhibited equivalent counts [*Z* = −1.091, *p* = not significant (NS)].

Kidney concerns referred to nine disorders: interstitial congestion or oedema, presence of melanomacrophage aggregates, glomerular atrophy, haemorrhage, tubular epithelium necrosis, tubular epithelium vacuolization, dilation of the glomerular capillaries, orange pigment in tubular epithelial cells and interstitial nephritis (Figure 3). Detailed description of histopathological alterations is presented in Supplementary Material. Concern severity varied 0–3 except for necrosis that varied 1–3. The overall score differed among groups (K–W: *χ* ^2^ = 8.850, *p* = 0.009). Post hoc M–W tests evidenced that lesioned and non‐lesioned DRE fishes had similar scores (groups 1 and 2: *Z* = −0.230, *p* = NS) higher than those in control Itapemirim specimens (groups 1 and 3: *Z* = −2.449, *p* = 0.014; groups 2 and 3: *Z* = −2.667, *p* = 0.008). Higher severity was detected in DRE (groups 1 and 2) for dilation, necrosis and melanomacrophages (M–W: 0.005 ≤ *p* ≤ 0.042), with lesioned specimens (group 1) also having higher atrophy and orange pigments (M–W: 0.013 ≤ *p* ≤ 0.030). Lower severity was evidenced for nephritis (M–W: 0.002 ≤ *p* ≤ 0.005).

### 3.4 Are fin lesions more prevalent in DRE fishes compared to control sites?

A total of 298 individuals of *G*. *genidens* were collected in the DRE (*N* = 93) and control sites (*N* = 85 for Itapemirim and *N* = 120 for São Mateus). Specimens' total length differed among sites (K–W test, *p* < 0.05), with means (and SD) of 234 (45.5 SD), 224 (24 SD) and 252 mm (26.4 SD) in the DRE, Itapemirim and São Mateus, respectively. Lesions in *G*. *genidens* were recorded only on caudal fins and showed a prevalence of 29% (*N* = 27) in the DRE and 0% in both control sites. Water parameters at the time of sampling were similar among sites. In the DRE, Itapemirim and São Mateus, respectively, mean salinity was low (0.0, 0.1 and 0.0 psu), mean temperature was equivalent (28.7, 24.6 and 27.7°C) and mean pH was near neutrality (7.1, 7.1 and 6.9).

Museum voucher specimens from the DRE represented 110 taxa (including specimens identified at the genus or family level) in 37 families. Of these, 38 taxa in 23 families presented damaged caudal fin (Table S2).

## 4 DISCUSSION

Our results indicate that piranhas, mainly *S*. cf. *brandtii*, are likely responsible for the observed fin lesions in DRE fishes, including *G. genidens*. Piranhas mostly live in shoals and exhibit a voracious piscivore habit. Particularly, *Serrasalmus* species are known to exhibit caudal fin‐nipping behaviour (i.e. small bites on the prey's caudal fin; Sazima & Pombal‐Jr, 1988; Sazima & Machado, 1990; Silva et al., 2015). Diet analysis confirmed that behaviour for *S*. cf. *brandtii* in the DRE, whereas *P. nattereri* act as a ‘classic’ predator. Similar to our findings, Assis et al. (2024) mentioned the presence of fish fins in the stomach contents of *S*. cf. *brandtii* in the freshwater area of the river, ~80 km upstream from the DRE. There, *S*. cf. *brandtii* inhabit mainly lakes and the river channel (Assis et al., 2024). In the DRE, 20 juvenile *S*. cf. *brandtii* were collected over the past 5 years through sieve and dip‐net sampling. Additionally, lakes in the vicinity of the DRE appear to support higher abundances of piranhas (*S*. cf. *brandtii* and *P*. *nattereri*) than the DRE itself. Seasonal flooding may play a crucial role in facilitating the movement of individuals from these adjacent lakes into the DRE. Whereas the introduction and ecological impact of *P*. *nattereri* in the DRB are well documented (Bueno et al., 2021; Fragoso‐Moura et al., 2016; Latini & Petrere, 2004), the occurrence of *S*. cf. *brandtii* is relatively recent, and its impacts are only now beginning to emerge. Assis et al. (2024) reported that, despite two sporadic records in 1987 and 2015, the consistent presence of *S*. cf. *brandtii* in the DRB was observed after 2020 – 1 year after the onset of extensive monitoring – and has increased substantially in the following years.

Our trials reveal that bacterial infections are associated with lesions observed on the caudal fin of the catfish *G. genidens*, with all lesioned caudal fins presenting some bacterial strains and lesioned fishes presenting more diverse and severe liver infections. However, because neither *Aeromonas*, *Pseudomonas* nor *Vibrio* strains were ubiquitous in caudal fins, we reject the fin rot disease hypothesis. That is, the most plausible scenario explaining our set of observations is an initial mutilation caused by piranha fin‐nipping that facilitates other bacterial attachment and progression to systemic infection. Bacterial colonization and infection would originate from the low‐quality DRE environment (including in unbitten individuals) or directly from the mouth of piranhas (Haddad & Sazima, 2003). Although *A. hydrophila* is known to infect wounds from piranha bite (Revord et al., 1988), that strain was exclusively detected in a few wound‐free DRE individuals. In sharp contrast, *Streptococcus* exhibited exclusive occurrence and 100% prevalence on fins of wounded individuals. Piranhas therefore appear to act as potent vectors for *Streptococcus* coinfection over environment‐driven primary bacterial infection, but this issue needs further investigation. As such, piranhas multiply their occupancy and predatory impact on already‐pollution‐stressed species by inflicting wounds that lead to additional bacterial infection. The ecological cost on native fish assemblages needs immediate evaluation.

According to our analysis, kidney disorders scored higher, that is, appeared in higher severity in DRE fish than in the control (Itapemirim River estuary) individuals, regardless of fin lesions. Therefore, the habitat seems to be the driver of organ disorders, not the fin‐nipping by piranhas. This hypothesis is supported by the observation that fish from the outer region of the DRE, well beyond the area of piranha occurrence, exhibit high levels of metal contamination and histopathological lesions similar to those we report (e.g. epithelial necrosis, interstitial congestion and cytoplasmic vacuolization) (Bevitório et al., 2022). Bacterial communities in the DRB, the river mouth and the marine area adjacent to it have also been impacted by the disaster (Almeida et al., 2023; Fernandes et al., 2022). Bacterial infections often prevail in waters under chronic or acute pollution conditions (Dar et al., 2020; Lindesjöö & Thulin, 1990; Mearns & Sherwood, 1974; Sherwood, 1978). Such long, sustained exposure to a depressed environment may contribute to an immunosuppressed and disease‐susceptible state that would offer a gateway for bacterial infections. Metal bioaccumulation from contaminated waters, as observed in DRE fishes (Gabriel et al., 2020), frequently leads to immune dysfunction, increasing the susceptibility of aquatic biota to infections (Dunier, 1996; Sauvé et al., 2002). In fact, the susceptibility of the potential host and the quality of the living environment are crucial in determining the odds of a disease spreading out (Dar et al., 2020). The high pathogen prevalence, bacterial strain diversity, individual bacterial co‐infestation (fin and liver) and occurrence of kidney disorders in DRE fish therefore indicate a potential bacterial outbreak.

## 5 CONCLUSIONS

Our results showed that fish with lesioned fins were found exclusively in the DRE where piranhas are present. Moreover, fin remains were identified in the stomach contents of all *S*. cf. *brandtii* individuals analysed from the DRE, reinforcing the role of piranhas as the likely agents of fin damage. Although bacterial infections and kidney disorders appear to be associated with the polluted conditions of the DRE, it is possible that piranha attacks contribute to or exacerbate secondary infections. Conceivably, wound‐colonizing bacteria from the mouth of piranhas or the environment could lead to further infections by other strains; then, either the initial or the additional infections become systemic (liver). In that respect, fin *Streptococcus* emerges as a strong indicator associated with fin lesion but does not appear to be internalized into liver infection. Therefore, healed fish cannot be diagnosed by bacterial screening. Only *C. diversu*s was detected in the liver and on fin in both DRE groups, but no single individual harboured *Citrobacter* in its liver and on fin simultaneously.

Regardless of the incidence of lesions, bacterial pathogens in the liver of DRE fish were more diverse and prevalent than those in the control area, and kidney disorders scored high for both DRE groups. This may indeed be attributed to the pollution status of the DRE, but further evaluation is needed to confirm this. The higher prevalence of systemic bacterial infections and the increased severity of organ damage in *G*. *genidens* from the DRE indicate that this population is experiencing poor health conditions in the polluted estuary. Additionally, individuals with fin lesions are likely subject to increased energetic costs, as fin damage can reduce swimming performance. This may, in turn, elevate predation risk, decrease foraging efficiency and potentially impair *G*. *genidens* social and reproductive behaviours, such as mouth‐brooding.

At the same time, a number of bacterial strains identified in our study are pathogenic in humans (Gauthier, 2015). Our results therefore draw attention to the possibility of zoonotic transmission via DRE fish, an issue that warrants further investigation to adequately address public health risks.

## AUTHOR CONTRIBUTIONS

**Ryan Andrades:** conceptualization, formal analysis, supervision, writing – original draft, writing – review and editing. **Helder C. Guabiroba:** conceptualization, data curation, investigation, methodology, writing – review and editing. **Kathiani V. Bastos:** data curation, investigation, methodology. **Vítor L. A. Rodrigues:** formal analysis, writing – review and editing. **Marcelo R. D. Santos:** resources, methodology. **Clarisse M. Arpini:** investigation, methodology, writing – review and editing. **Mayra C. Flecher:** investigation, methodology, writing – review and editing. **Helen A. Pichler:** resources, writing – review and editing. **Ciro V. Vilar:** writing – review and editing. **Maurício Hostim‐Silva:** resources, project administration. **Jean‐C. Joyeux:** resources, project administration, supervision, writing – review and editing.

## FUNDING INFORMATION

Renova Foundation.

## CONFLICT OF INTEREST STATEMENT

The authors declare the following financial interests/personal relationships which may be considered as potential competing interests. Ryan Andrades, Helder C. Guabiroba, Kathiani V. Bastos, Vítor L. A. Rodrigues, Helen A. Pichler, Ciro C. Vilar, Maurício Hostim‐Silva and Jean‐Christophe Joyeux report a relationship with the Renova Foundation that includes funding grants. Currently the following authors receive or have received a fellowship (Ryan Andrades, Ciro C. Vilar, Helen A. Pichler, Maurício Hostim‐Silva and Jean‐Christophe Joyeux) or salary (Helder C. Guabiroba, Vítor L. A. Rodrigues and Kathiani V. Bastos) from the Espírito Santo Foundation for Technology (FEST: Fundação Espírito‐Santense de Tecnologia, http://www.fest.org.br/fest2018/) for their participation in the Aquatic Biodiversity Monitoring Program (PMBA: Programa de Monitoramento da Biodiversidade Aquática) conducted as part of an agreement between FEST and the Renova Foundation. The Renova Foundation is responsible for reparation of damages caused by the collapse of the Fundão dam in Mariana (MG, Brazil). Neither the FEST, PMBA or Renova Foundation played any role in the interpretation of the results or preparation of the manuscript. If there are other authors, they declare that they have no known competing financial interests or personal relationships that could have appeared to influence the work reported in this paper.

## Supporting information


**Table S1.** Phenotypic characterization of isolated bacteria and fungus. Oxydase, Catalase, and Motility: absence (−), presence (+). NA = not applicable, NO = not observed.
**Table S2.** Fish taxa with mutilated caudal fin in the Doce river estuary.

## Data Availability

Part of the data supporting the findings of this study are included in this article and its Supplementary Material. The raw datasets generated and/or analysed during the current study are available from the corresponding author (Ryan Andrades) upon reasonable request.
