# Supplementary material for: The emergence of pathogens on fish in an impacted estuary and the role of non‐native piranhas in a potential bacterial infectious outbreak
Source: J Fish Biol. 2026 Mar 20;108(5):1515–23. doi: 10.1111/jfb.70377 (PMC13273093; doi:10.1111/jfb.70377)
Supplement: Supplementary file 1 — Table S1. Phenotypic characterization of isolated bacteria and fungus. Oxydase, Catalase, and Motility: absence (−), presence (+). NA = not applicable, NO = not observed. Table S2. Fish taxa with mutilated caudal fin in the Doce river estuary. [file JFB-108-1515-s001.docx]

**JFB-MS-24-0937**

Supplementary Material

The emergence of pathogens on fish in an impacted estuary and the role of non-native piranhas in a potential bacterial infectious outbreak

R. Andrades et al.

This file includes:

- Description of histopathological alterations of *Genidens genidens* individuals;

- Table S1: Phenotypic characterization of isolated bacteria and fungus;

- Table S2: Fish taxa with mutilated caudal fin in the Doce river estuary.

**Description of histopathological alterations of *Genidens genidens* individuals. GGFR (*G*. *geniden*s with fin lesions from Doce river estuary), GGRD (*G*. *geniden*s without fin lesions from Doce river estuary), and GGIT (*G*. *geniden*s without fin lesions from Itapemirim river estuary).**

GGFR 01: Moderate multifocal vacuolization (degeneration) and moderate multifocal tubular epithelial necrosis, moderate multifocal glomerular capillary dilation and mild multifocal glomerular atrophy. Large amount of multifocal brown pigments forming clusters (melanomacrophages), and diffuse marked mononuclear interstitial nephritis.

GGFR 02: Moderate interstitial congestion, moderate multifocal vacuolization (degeneration) and moderate multifocal tubular epithelial necrosis, marked multifocal glomerular capillary dilation. Large amount of multifocal brown pigments forming clusters (melanomacrophages), orange pigment in mild multifocal tubular epithelial cells, and diffuse marked mononuclear interstitial nephritis.

GGFR 03: Moderate multifocal vacuolization (degeneration) and marked multifocal tubular epithelial necrosis, marked interstitial edema, and diffuse moderate mononuclear interstitial nephritis. Marked atrophy of glomeruli.

GGFR 04: Mild multifocal interstitial congestion, moderate multifocal vacuolization (degeneration) and marked multifocal tubular epithelial necrosis, moderate glomerular atrophy. Large amount of multifocal brown pigments forming clusters (melanomacrophages), orange pigment in multifocal tubular epithelial cells marked. Diffuse moderate mononuclear interstitial nephritis.

GGFR 05: Mild multifocal interstitial congestion, moderate multifocal vacuolization (degeneration) and marked multifocal tubular epithelial necrosis, moderate glomerular atrophy. Large amount of multifocal brown pigments forming clusters (melanomacrophages), orange pigment in multifocal tubular epithelial cells marked. Diffuse moderate mononuclear interstitial nephritis.

GGFR 06: Marked interstitial congestion, moderate multifocal vacuolization (degeneration) and mild multifocal tubular epithelial necrosis, marked multifocal glomerular capillary dilation. Large amount of multifocal brown pigments forming clusters, and diffuse marked mononuclear interstitial nephritis.

GGFR 07: Marked interstitial congestion, moderate hemorrhage, moderate multifocal vacuolization (degeneration) and moderate multifocal tubular epithelial necrosis, mild multifocal glomerular capillary dilation. Large amount of multifocal brown pigment forming clusters (melanomacrophages), and orange pigment in mild multifocal tubular epithelial cells.

GGFR 08: Marked interstitial congestion, moderate hemorrhage, moderate multifocal vacuolization (degeneration) and moderate multifocal tubular epithelial necrosis, mild multifocal glomerular capillary dilation. Large amount of multifocal brown pigment forming clusters (melanomacrophages), orange pigment in mild multifocal tubular epithelial cells and mild to moderate diffuse mononuclear interstitial nephritis.

GGFR 09: Mild multifocal interstitial congestion, mild multifocal vacuolization (degeneration) and necrosis of mild multifocal tubular epithelium, diffuse moderate mononuclear interstitial nephritis and an amount of multifocal brown pigments forming clusters (melanomacrophages).

GGFR 10: Mild multifocal interstitial congestion, mild multifocal vacuolization (degeneration) and necrosis of mild multifocal tubular epithelium, diffuse moderate mononuclear interstitial nephritis and an amount of multifocal brown pigments forming clusters (melanomacrophages).

GGRD 01: Moderate multifocal vacuolization (degeneration) and necrosis of the multifocal tubular epithelium and moderate multifocal dilation of the glomerular capillaries. Large amount of multifocal brown pigments forming clusters (melanomacrophages) and diffuse marked mononuclear interstitial nephritis.

GGRD 02: Moderate interstitial congestion, moderate multifocal vacuolization (degeneration) and moderate multifocal tubular epithelial necrosis, marked multifocal glomerular capillary dilation. Large amount of multifocal brown pigments forming clusters (melanomacrophages), orange pigment in mild multifocal tubular epithelial cells and diffuse marked mononuclear interstitial nephritis.

GGRD 03: Moderate multifocal vacuolization (degeneration) and marked multifocal tubular epithelial necrosis, marked interstitial edema, diffuse moderate mononuclear interstitial nephritis. Marked atrophy of glomeruli.

GGRD 04: Mild multifocal interstitial congestion, moderate multifocal vacuolization (degeneration) and marked multifocal tubular epithelial necrosis, moderate glomerular atrophy. Large amount of multifocal brown pigments forming clusters (melanomacrophages), orange pigment in multifocal tubular epithelial cells marked. Diffuse moderate mononuclear interstitial nephritis.

GGRD 05: Mild multifocal interstitial congestion, moderate multifocal vacuolization (degeneration) and moderate multifocal tubular epithelial necrosis, diffuse moderate mononuclear interstitial nephritis and large amounts of multifocal brown pigments forming clusters (melanomacrophages).

GGRD 06: Marked interstitial congestion, moderate multifocal vacuolization (degeneration) and mild multifocal tubular epithelial necrosis, marked multifocal glomerular capillary dilation. Large amount of multifocal brown pigments forming clusters and diffuse marked mononuclear interstitial nephritis.

GGRD 07: Marked interstitial congestion, moderate hemorrhage, moderate multifocal vacuolization (degeneration) and moderate multifocal tubular epithelial necrosis, mild multifocal glomerular capillary dilation. Large amount of multifocal brown pigment forming clusters (melanomacrophages), and orange pigment in mild multifocal tubular epithelial cells.

GGRD 08: Marked interstitial congestion, moderate hemorrhage, moderate multifocal vacuolization (degeneration) and moderate multifocal tubular epithelial necrosis, mild multifocal glomerular capillary dilation. Large amount of multifocal brown pigment forming clusters (melanomacrophages), orange pigment in mild multifocal tubular epithelial cells, and mild to moderate diffuse mononuclear interstitial nephritis.

GGRD 09: Marked interstitial congestion, moderate hemorrhage, moderate multifocal vacuolization (degeneration) and moderate multifocal tubular epithelial necrosis, mild multifocal glomerular capillary dilation. Large amount of multifocal brown pigment forming clusters (melanomacrophages), orange pigment in mild multifocal tubular epithelial cells, and mild to moderate diffuse mononuclear interstitial nephritis.

GGRD 10: Marked interstitial congestion, moderate hemorrhage, moderate multifocal vacuolization (degeneration) and moderate multifocal tubular epithelial necrosis, mild multifocal glomerular capillary dilation. Large amount of multifocal brown pigment forming clusters (melanomacrophages), orange pigment in mild multifocal tubular epithelial cells, and mild to moderate diffuse mononuclear interstitial nephritis.

GGIT 01: Diffuse marked mononuclear interstitial nephritis, moderate multifocal vacuolization (degeneration) and mild multifocal tubular epithelial necrosis. Mild multifocal brown pigments forming clusters (melanomacrophages).

GGIT 02: Mild congestion, diffuse marked mononuclear interstitial nephritis, mild multifocal vacuolization (degeneration) and mild multifocal tubular epithelial necrosis. Mild multifocal brown pigments forming clusters (melanomacrophages).

GGIT 03: Diffuse marked mononuclear interstitial nephritis, marked multifocal vacuolization (degeneration) and moderate multifocal tubular epithelial necrosis. Moderate multifocal brown pigments forming clusters (melanomacrophages).

GGIT 04: Mild congestion, diffuse marked mononuclear interstitial nephritis, mild multifocal vacuolization (degeneration) and mild multifocal tubular epithelial necrosis. Mild multifocal brown pigments forming clusters (melanomacrophages).

GGIT 05: Moderate congestion, marked diffuse mononuclear interstitial nephritis, marked multifocal vacuolization (degeneration) and mild multifocal tubular epithelial necrosis. Moderate multifocal brown pigments forming clusters (melanomacrophages).

GGIT 06: Moderate congestion, moderate hemorrhage, marked diffuse mononuclear interstitial nephritis, marked multifocal brown pigments forming clusters (melanomacrophages), moderate multifocal vacuolization (degeneration) and moderate multifocal tubular epithelial necrosis.

GGIT 07: Moderate congestion, moderate hemorrhage, marked diffuse mononuclear interstitial nephritis, mild multifocal vacuolization (degeneration) and mild multifocal tubular epithelium necrosis. Mild multifocal brown pigments forming clusters (melanomacrophages).

GGIT 08: Diffuse marked mononuclear interstitial nephritis, mild multifocal vacuolization (degeneration) and necrosis of mild multifocal tubular epithelium. Mild multifocal brown pigments forming clusters (melanomacrophages).

GGIT 09: Diffuse marked mononuclear interstitial nephritis, mild multifocal vacuolization (degeneration) and necrosis of mild multifocal tubular epithelium. Mild multifocal brown pigments forming clusters (melanomacrophages).

GGIT 10: Moderate congestion, moderate hemorrhage, marked diffuse mononuclear interstitial nephritis, marked multifocal brown pigments forming clusters (melanomacrophages), moderate multifocal vacuolization (degeneration) and moderate multifocal tubular epithelial necrosis.

Table S1: Phenotypic characterization of isolated bacteria and fungus. Oxydase, Catalase, and Motility: absence (-), presence (+). NA=not applicable, NO=not observed.

| Strain | Gram | Morphology | Oxidase | Catalase | Motility | Fermentation | Observations |  | |  | |  |
| --- | --- | --- | --- | --- | --- | --- | --- | --- | --- | --- | --- | --- |
| **BACTERIA** |  |  |  |  |  |  |  |  | |  | |  |
| *Pseudomonas aeruginosa* | Negative | Bacillus | + | + | + | Lactose -, Glucose - | Pyocyanin production, growth on cetrimide agar, arginine +, lipase +, mannitol + | | | | | |
| *Klebsiella ornithinolytica* | Negative | Bacillus | - | + | - | Lactose - | Urease+, Voges-Proskauer+, indole+, ornithine decarboxylase+ | | | | | |
| Actinomycetota | Positive | Branched filamentous | - | + | - | NO |  |  | |  | |  |
| *Aeromonas hydrophila* | Negative | Bacillus | + | + | + | Glucose + | Beta-hemolysis, indole +, Voges-Proskauer +, H2S +, arginine + | | | | | |
| *Citrobacter diversus* | Negative | Bacillus | - | + | + | Lactose + | Indole +, urease +, H2S -, mannitol +, sorbitol + | | | | | |
| *Citrobacter diversus* (*koseri*) | Negative | Bacillus | - | + | + | Lactose + | Indole +, urease +, H2S -, Voges-Proskauer -, mannitol+, sorbitol+ | | | | | |
| *Citrobacter freundii* | Negative | Bacillus | - | + | + | Lactose + | H2S +, indole -, urease +, glucose +, mannitol +, sorbitol +, maltose + | | | | | |
| *Enterobacter cloacae* | Negative | Bacillus | - | + | + | Lactose + | Voges-Proskauer +, indole -, urease +, glucose +, mannitol +, sorbitol + | | | | | |
| *Enterococcus faecalis* | Positive | coccus in chain | - | - | - | Glucose +, Esculin + | Gamma hemolysis, cNaCl 6.5% negative growth, catalase negative | | | | | |
| *Escherichia coli* | Negative | Bacillus | - | + | + | Lactose + | Indole +, gas +, glucose acid +, characteristic growth in EMB | | | | | |
| *Proteus vulgaris* | Negative | Bacillus | - | + | +++ | Glucose + (gas production) | H2S +, urease +, indole +, deamine tryptophan +, swarming in agar + | | | | | |
| *Pseudomonas aeruginosa* | Negativo | Bacillus | + | + | + | Lactose -, Glucose - | Pyocyanin production, growth on cetrimide agar, arginine +, lipase +, mannitol + | | | | | |
| *Pseudomonas luteola* | Negative | Bacillus | - | + | + | Lactose -, Glucose - | arginine -, mannitol +, urease - | | | |  | |
| *Rhodococcus rhodochrous* | Positive | coccobacillus | - | + | - | Oxidative | Production of red pigment | |  | |  | |
| *Streptococcus* sp. | Positive | coccus in chain | - | - | - | Esculin + | Alpha hemolysis, NaCl 6.5% negative growth, catalase negative | | | | | |
| **FUNGUS** |  |  |  |  |  |  |  |  | |  | |  |
| *Aspergillus* sp. | NA | Filamentous fungus | NA | NA | NA | NA | Green colonies, conidiophores with vesicles, septate hyphae | | | | | |

Table S2: Fish taxa with mutilated caudal fin in the Doce river estuary.

| CLUPEIFORMES |  |  |
| --- | --- | --- |
|  | Dorosomatidae |  |
|  |  | *Lile piquitinga* (Schreiner & Miranda Ribeiro, 1903) |
|  | Engraulidae |  |
|  |  | *Lycengraulis grossidens* (Spix & Agassiz, 1829) |
| CHARACIFORMES |  |  |
|  | Erythrinidae |  |
|  |  | *Hoplias intermedius* (Günther, 1864) |
|  | Serrasalmidae |  |
|  |  | *Serrasalmus* cf. *brandtii* Lütken, 1875 |
|  | Bryconidae |  |
|  |  | *Salminus brasiliensis* (Cuvier, 1816) |
|  | Characidae |  |
|  |  | *Astyanax* aff. *intermedius* (Eigenmann, 1908) |
|  |  | *Astyanax* cf. *bimaculatus* (Linnaeus, 1758) |
| SILURIFORMES |  |  |
|  | Loricariidae |  |
|  |  | *Hypostomus scabriceps* (Eigenmann & Eigenmann, 1888) |
|  | Ariidae |  |
|  |  | *Cathorops spixii* (Agassiz, 1829) |
|  |  | *Genidens barbus* (Lacepède, 1803) |
|  |  | *Genidens genidens* (Cuvier, 1829) |
|  |  | *Paragenidens grandoculis* (Steindachner 1877) |
|  | Pseudopimelodidae |  |
|  |  | *Microglanis pataxo* Sarmento-Soares, Martins-Pinheiro, Aranda & Chamon 2006 |
| SYNGNATHIFORMES |  |  |
|  | Syngnathidae |  |
|  |  | *Microphis lineatus* (Kaup, 1856) |
| GOBIIFORMES |  |  |
|  | Eleotridae |  |
|  |  | *Dormitator maculatus* (Bloch, 1792) |
|  |  | *Eleotris pisonis* (Gmelin, 1789) |
|  | Gobiidae |  |
|  |  | *Awaous tajasica* (Lichtenstein, 1822) |
|  |  | *Ctenogobius apogonus* Pezold, 2022 |
|  |  | *Evorthodus lyricus* (Girard, 1858) |
| MUGILIFORMES |  |  |
|  | Mugilidae |  |
|  |  | *Mugil curema* Valenciennes, 1836 |
| CICHLIFORMES |  |  |
|  | Cichlidae |  |
|  |  | *Coptodon rendalli* (Boulenger, 1897) |
|  |  | *Crenicichla* cf. *lacustris* (Castelnau, 1855) |
|  |  | *Geophagus brasiliensis* (Quoy & Gaimard, 1824) |
|  |  | *Oreochromis* cf. *niloticus* (Linnaeus, 1758) |
| ATHERINIFORMES |  |  |
|  | Atherinopsidae |  |
|  |  | *Atherinella brasiliensis* (Quoy & Gaimard, 1825) |
| CYPRINODONTIFORMES |  |  |
|  | Poeciliidae |  |
|  |  | *Poecilia vivipara* Bloch & Schneider, 1801 |
| CARANGIFORMES |  |  |
|  | Carangidae |  |
|  |  | *Caranx* sp. |
| CARANGIFORMES |  |  |
|  | Achiridae |  |
|  |  | *Trinectes paulistanus* (Miranda Ribeiro, 1915) |
|  | Centropomidae |  |
|  |  | *Centropomus parallelus* Poey, 1860 |
|  |  | *Centropomus undecimalis* (Bloch, 1792) |
| GERREIFORMES |  |  |
|  | Gerreidae |  |
|  |  | *Diapterus auratus* Ranzani, 1842 |
|  |  | *Eucinostomus argenteus* Baird & Girard, 1855 |
|  |  | *Eucinostomus melanopterus* (Bleeker, 1863) |
|  |  | *Eugerres brasilianus* (Cuvier, 1830) |
| EUPERCARIA *incertae sedis* |  |  |
|  | Lutjanidae |  |
|  |  | *Lutjanus jocu* (Bloch & Schneider, 1801) |
|  | Sciaenidae |  |
|  |  | *Micropogonias furnieri* (Desmarest, 1823) |
|  |  | *Pachyurus adspersus* Steindachner, 1879 |
| TETRAODONTIFORMES |  |  |
|  | Tetraodontidae |  |
|  |  | *Sphoeroides testudineus* (Linnaeus, 1758) |
